# The Role of Von Willebrand Factor in the Pathogenesis of Pulmonary Vascular Thrombosis in COVID-19

**DOI:** 10.3390/v14020211

**Published:** 2022-01-21

**Authors:** Anastasiya S. Babkina, Irina V. Ostrova, Mikhail Ya Yadgarov, Artem N. Kuzovlev, Andrey V. Grechko, Alexey V. Volkov, Arkady M. Golubev

**Affiliations:** 1 Federal Research and Clinical Center of Intensive Care Medicine and Rehabilitology, 107031 Moscow, Russia; iostrova@fnkcrr.ru (I.V.O.); mikhail.yadgarov@mail.ru (M.Y.Y.); artem_kuzovlev@fnkcrr.ru (A.N.K.); avg-2007@ya.ru (A.V.G.); alex.volkoff@gmail.com (A.V.V.); arkadygolubev@mail.ru (A.M.G.); 2 Department of Forensic Medicine, Institute of Medicine, Peoples’ Friendship University of Russia, 117198 Moscow, Russia; 3 Department of Pathological Anatomy, Institute of Medicine, Peoples’ Friendship University of Russia, 117198 Moscow, Russia

**Keywords:** SARS-CoV-2, COVID-19, ARDS, von Willebrand factor, thrombosis, pulmonary embolism

## Abstract

The increased plasma levels of von Willebrand factor (VWF) in patients with COVID-19 was reported in many studies, and its correlation with disease severity and mortality suggest its important role in the pathogenesis of thrombosis in COVID-19. We performed histological and immunohistochemical studies of the lungs of 29 patients who died from COVID-19. We found a significant increase in the intensity of immunohistochemical reaction for VWF in the pulmonary vascular endothelium when the disease duration was more than 10 days. In the patients who had thrombotic complications, the VWF immunostaining in the pulmonary vascular endothelium was significantly more intense than in nonsurvivors without thrombotic complications. Duration of disease and thrombotic complications were found to be independent predictors of increased VWF immunostaining in the endothelium of pulmonary vessels. We also revealed that bacterial pneumonia was associated with increased VWF staining intensity in pulmonary arterial, arteriolar, and venular endothelium, while lung ventilation was an independent predictor of increased VWF immunostaining in arterial endothelium. The results of the study demonstrated an important role of endothelial VWF in the pathogenesis of thrombus formation in COVID-19.

## 1. Introduction

The novel coronavirus infection (COVID-19) pandemic caused by SARS-CoV-2, an RNA-containing virus of the family Coronaviridae, genus Betacoronavirus, has claimed more than 5 million lives to date [1]. Despite significant progress in the treatment of the disease, several issues concerning the pathogenesis of COVID-19 remain open. The predominantly respiratory clinical manifestations of COVID-19, morphological evidence of lung damage, and respiratory failure as the most frequent cause of death suggest that the lungs are the main target organ for the SARS-CoV-2 virus.

Morphological study of internal organs of patients who died of COVID-19 shows damage and circulatory disturbances in the lungs (corresponding to acute respiratory distress syndrome) and other organs [2,3].

The reported complications of COVID-19 indicate the involvement of the hemostatic, central nervous, cardiovascular, and genitourinary systems in the disease mechanisms [4,5,6,7]. One of the possible mechanisms contributing to extrapulmonary complications in COVID-19 is viremia, which has been identified in patients with severe infection in several studies [8,9].

However, whether these complications result from the cytopathic effects of the SARS-CoV-2 virus or are due to hypoxia associated with respiratory failure is still unclear. Besides, the role of comorbidities and iatrogenic complications in the pathogenesis of disease and death mechanisms in COVID-19 cannot be excluded.

As thrombotic complications are often fatal, coagulopathy should be considered along with respiratory failure as the most important aspect of COVID-19 pathogenesis. Thrombotic complications, including venous and arterial thrombosis, thromboembolism, and disseminated intravascular coagulation, are known to develop in a third of critically ill COVID-19 patients in the intensive care unit [10].

Hemostatic disorders have been described in acute respiratory distress syndrome (ARDS), acute lung injury of various etiologies, as well as in many viral diseases, including coronavirus infection, Ebola and Dengue fever [11,12,13,14].

The hypothesis that advanced coronavirus infection is characterized primarily by progressive endothelial damage causing coagulation disorders has been confirmed in many studies [15]. Spadaro et al. showed that plasma levels of endothelial damage markers such as angiopoietin-2 (ANG-2), intercellular adhesion molecule-1 (ICAM-1), vascular cell adhesion molecule-1 (VCAM-1), P-selectin, and E-selectin were higher in patients with ARDS caused by COVID-19 than in patients with ARDS of other etiologies [16]. The progression of endothelial damage was evidenced by the study of Tojo et al., which demonstrated an increase in ANG-2 levels in advanced disease, in contrast to those of soluble receptor for advanced glycation end products (sRAGE), a marker of alveolar endothelial damage, which rose on the first day of hospitalization and gradually decreased throughout the hospital stay [17]. Morphological examination revealed endothelial damage, particularly in pulmonary arteries and arterioles, consisting of endothelial cell desquamation and cytoplasmic vacuolization [18,19].

Two main mechanisms of endothelial damage are suggested: (1) direct endothelial damage by SARS-CoV-2 virus; and (2) endothelial damage due to hyperproduction of cytokines, chemokines, complement system activation, and intense NETosis, which leads to impaired endothelial antithrombogenic properties and activation of prothrombotic factors, in particular, VWF [20].

Increased plasma VWF levels in patients with COVID-19 were observed in many studies, and their correlation with the disease severity and mortality suggests their important role in the pathogenesis of thrombosis in COVID-19 [21]. The high VWF level has been shown to associate with an adverse clinical outcome in acute lung injury and ARDS [22].

However, in most studies, the plasma levels of VWF were measured, which did not allow objective assessment of the role of endothelial VWF in the thrombosis development in COVID-19. The changes in endothelial activation markers, including VWF, throughout the disease have not been fully investigated. The morphological study of lungs in patients who died of COVID-19 using immunohistochemical staining for VWF may be helpful for a better understanding of the pathogenesis of thrombosis in this disease.

Objective: to provide a rationale for the role of pulmonary vascular endothelial VWF in thrombus formation in COVID-19.

## 2. Materials and Methods

Autopsy reports and lung autopsy materials of 29 patients who died in October 2020 with confirmed novel coronavirus infection were analyzed. The study included fatal cases with COVID-19 as the primary disease. The direct causes of death were respiratory failure in 28 cases and massive pulmonary embolism in 1 case. Exclusion criteria was a delay of more than 2 days after death before postmortem examination.

The autopsies were done in the pathological anatomy department of the Mukhin Municipal Clinical Hospital of the Moscow Department of Health Care. Organ specimens for histological examination were taken in accordance with the current legislation of the Russian Federation to verify the final diagnosis and the cause of death. The study protocol was approved by the local ethics committee (№ 2/21/3). In accordance with the current Guidelines of the Ministry of Health of the Russian Federation, the organ specimens were fixed for 72 h in a neutral 10% formalin solution [23]. Formalin-fixed, paraffin-embedded tissues underwent standard processing to provide sections stained with hematoxylin and eosin.

The histochemical stain phosphotungstic acid–hematoxylin (PTAH) was used for fibrin detection. Weigert–van Gieson staining was used to detect elastic fibers, connective tissue, and collagen. Histological preparations were examined using a Nikon Eclipse Ni-U microscope (Nikon, Tokyo, Japan).

For immunohistochemical (IHC) study, the sections were deparaffinized in xylene and successively dehydrated in alcohol. High-temperature antigen unmasking was performed in citrate buffer, pH 6 (Target Retrieval Solution, DAKO, Glostrup, Denmark). Sections were cooled, washed 3 times in distilled water, placed in immunostaining chambers (Shandon Coverplate, Thermoscientific, Runcorn, UK), and washed with phosphate buffer (3 × 5 min) (PBS IHC Wash Buffer + Tween, Cell Marque, Rocklin, CA, USA). To suppress endogenous peroxidase activity, the sections were incubated in 3% hydrogen peroxide solution for 10 min. To prevent nonspecific binding of primary or secondary antibodies to tissue proteins, a blocking serum (Protein Block Serum-free, Abcam, Cambridge, UK) was used for 15 min. The sections were then incubated for 1 h at 37 °C with primary polyclonal antibodies against VWF (ab9378, 1:50 dilution in Antibody Diluent ab64211, Abcam, Cambridge, UK). Sections were washed with PBS (2 × 5 min). The intensity of the reaction was detected using the Mouse and Rabbit Specific HRP/DAB (ABC) Detection IHC kit (ab64264, Abcam, Cambridge, UK). After washing the sections in PBS, they were counterstained with hematoxylin. After washing in tap water, the slides were prepared using Immu-Mount water-soluble medium (Thermo Shandon, Pittsburgh, PA, USA).

After receiving digital images (10–20 images from each preparation, ×400), the intensity of IHC staining to VWF was determined using the mean optical density of the pulmonary vascular (arterial, arteriolar, venous, and venular) endothelium by the Nis-Elements BR image analysis software (Nikon, Tokyo, Japan) (Figure 1).

Statistical analysis was done using the SPSS Statistics 25.0 software. Normality of data distribution was assessed using the Shapiro–Wilk and Kolmogorov–Smirnov tests with a Lilliefors correction. All patients were divided into groups: patients with duration of illness more/less than 10 days (n = 17/12, respectively); patients with thrombotic complications (n = 15) including pulmonary artery embolism (n = 7) and pulmonary artery thrombosis (n = 8), and without thrombotic complications (n = 14); patients older than/under 57 years (n = 15/14, respectively); with/without ventilatory support (n = 19/10); with/without bacterial pneumonia (n = 8/21); female/male sex (9/20) (Supplementary Materials). Cut-off points were selected based on the median value of the parameter in the sample. The duration of the disease was the sum of the length of hospital stay (from admission to death) and the number of days from the onset of symptoms to admission according to the history. Patients with an established source of thromboembolism were assigned to the group of patients with pulmonary artery embolism. Comparative intergroup analysis was performed using nonparametric Mann–Whitney U-criterion for quantitative variables and Fisher’s exact test for frequencies (univariate statistical analysis). The critical level of significance was set at 0.05. Data are presented as Me (IQR), where Me is median value and IQR is the interquartile range.

To identify factors independently influencing the intensity of VWF immunostaining in the pulmonary vascular endothelium, we performed a multivariable analysis based on binary logistic regression, with estimation of the adjusted odds ratio (adj. OR) and its 95% confidence interval (CI). A stepwise inverse method (likelihood ratio) was used to include predictors in the regression model. We considered the following as potential factors influencing the increased intensity of VWF immunostaining in endothelium: duration of the disease (day 10 and more), thrombotic complications, age (57 years and older), ventilatory support, bacterial pneumonia, female sex.

## 3. Results

### 3.1. Morphological Study of the Lungs

**Figure 2:**
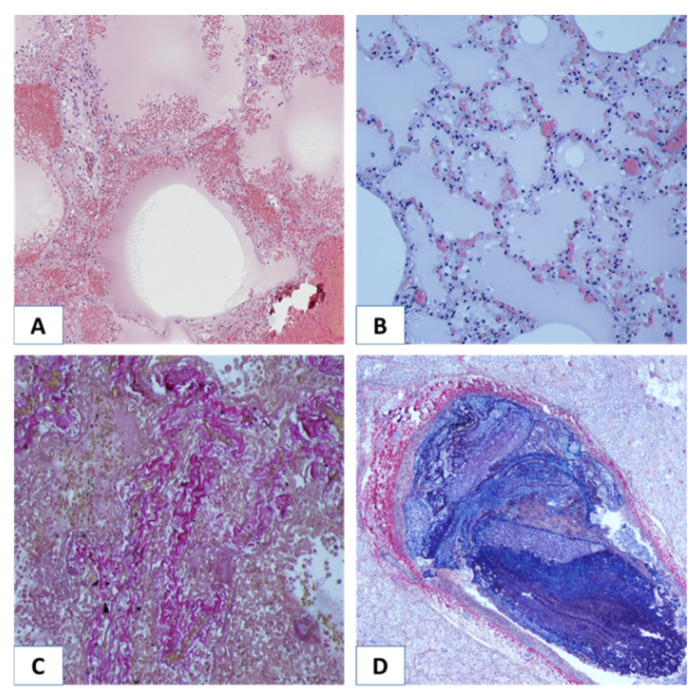
Morphological alterations in lungs of COVID-19 nonsurvivors. (**A**) Hyaline membranes and alveolar edema. Hematoxylin and eosin staining, ×100 magnification. (**B**) Alveolar edema. Hematoxylin and eosin staining, ×100. (**C**) Lung fibrosis. Elastic fibers were stained purple-red to brown. Collagen was stained various shades of red. Weigert–van Gieson staining, ×200. (**D**) Thrombus in the pulmonary artery. PTAH staining, ×40 magnification.

Morphological study of lungs of COVID-19 nonsurvivors revealed changes typical of ARDS. At all stages of the disease, lung alterations (desquamation of bronchial and alveolar epithelium), circulatory disturbances (alveolar edema, hyaline membranes, alveolar hemorrhages, microcirculatory congestion, pulmonary vascular thrombi), along with compensatory and adaptive processes (such as fibrosis) were registered (Figure 2). We found that the lung morphological changes in COVID-19 did not correlate with the stages of ARDS. Thus, fibrosis, which is not typical for the first (exudative) stage of ARDS, was detected in 50% of the patients with a disease duration of less than 10 days, which may have been due to late hospitalization or a specific pattern of fibrosis development in COVID-19. Alveolar edema, typical for the first week of ARDS, was detected in more than half of the patients after 10 days of hospitalization. In one-third of patients (8 out of 29), purulent pneumonia in the lungs was detected, indicating bacterial infection.

Patients who died before day 10 of the disease (median disease duration) had thrombotic complications in 33% of cases (4 patients out of 12). In patients with disease duration longer than 10 days, the percentage of thrombotic complications increased to 65% (11 patients out of 17); the differences were not significant (*p* = 0.139). Thrombotic complications included pulmonary embolism (PE) and pulmonary vascular thrombosis. A trend toward an increased incidence of pulmonary vascular thrombosis was recorded with disease duration of 10 days or more (*p* = 0.093). Pulmonary vascular thrombosis accounted for a major part of thrombotic complications in patients with disease duration of more than 10 days (Table 1).

### 3.2. Immunohistochemistry Study of VWF in the Pulmonary Vascular Endothelium of COVID-19 Nonsuvivors

**Figure 3:**
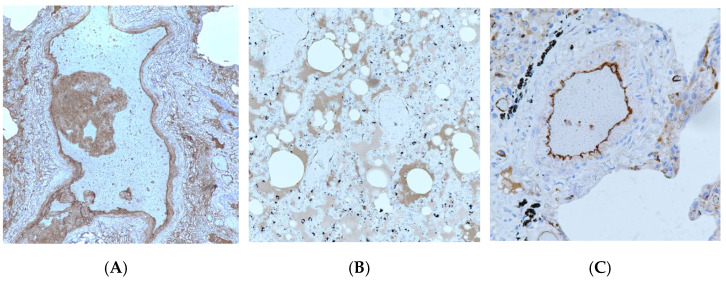
Positive VWF immunostaining: (**A**) in the mural thrombus, ×100 magnification; (**B**) in the edematous fluid in the alveoli, ×200 magnification; (**C**) in the pulmonary vascular endothelium, ×400 magnification.

Positive immunoreaction for VWF was found in the endothelium of large and microcirculatory vessels, edematous fluid, and thrombi (Figure 3).

**Figure 4:**
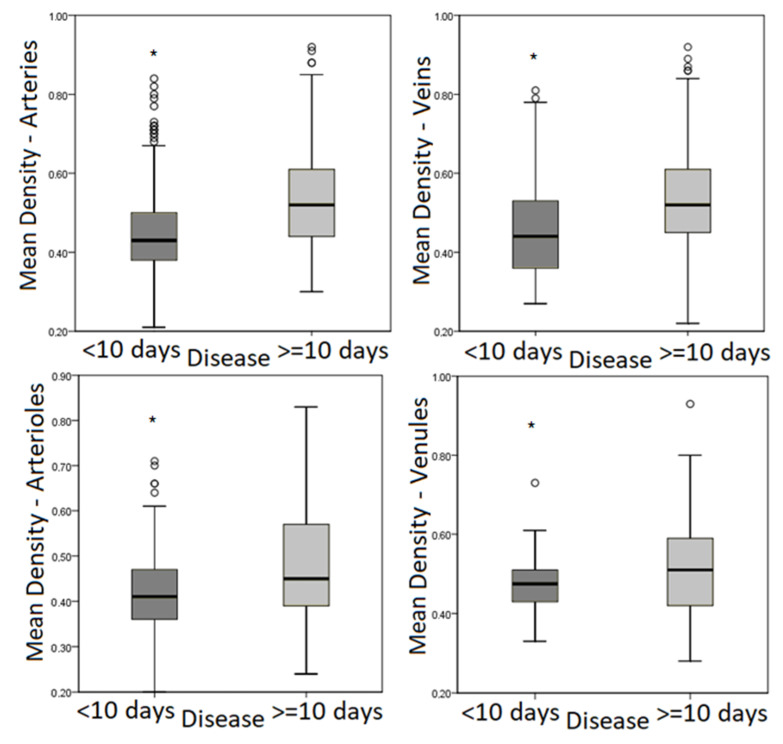
Box plot diagram showing distribution of median values and quartiles of mean optical density in the pulmonary vascular endothelium in groups of patients with disease duration of more/less than 10 days. Note: * significant differences.

A comparative intergroup analysis revealed that patients with disease duration of 10 days or more had significantly higher mean optical density of VWF staining in the endothelium of arteries, veins, arterioles, and venules (Table 2, Figure 4).

Patients with thrombotic complications (PE and/or thrombosis) had significantly higher mean optical density in the endothelium of veins and arteries compared to patients without thrombotic complications (Table 3).

Patients with thrombosis had significantly higher mean optical density in the endothelium of arteries, veins, and arterioles than patients with PE (Table 4).

### 3.3. Analysis of Independent Factors Associated with an Increase in Von Willebrand Factor in Pulmonary Vascular Endothelium

*Arteries.* Increased mean optical density in arterial endothelium by multivariable analysis was associated with disease duration of more than 10 days, thrombotic complications, ventilatory support, and bacterial pneumonia (Table 5).

*Veins.* Increased mean optical density in the venous endothelium was associated with disease duration of more than 10 days, thrombotic complications, and female sex (Table 6).

*Arterioles.* Increased mean optical density in the endothelium of arterioles was associated with a disease duration of more than 10 days, thrombotic complications, and bacterial pneumonia (Table 7).

*Venules.* Increased optical density in venule endothelium was associated with bacterial pneumonia and female sex (Table 8).

## 4. Discussion

Increased intensity of VWF immunostaining in pulmonary endothelium, a trend toward increasing rate of pulmonary arterial thrombosis in patients with disease duration of more than 10 days, supported the hypothesis regarding progression of circulatory disorders across the entire period of the disease. The important role of VWF in the pathogenesis of thrombosis in COVID-19 was proved by significantly higher intensity of IHC reaction in pulmonary vascular endothelium of patients with thrombosis compared to patients with PE. Several studies showed the association of high blood VWF levels with arterial thrombosis [24]. Kim et al., using simulation of arterial thrombosis in silico, in vitro, and in vivo, demonstrated an important role of VWF present in α-granules of platelets in the development of occlusive arterial thrombosis [25]. However, the results of published studies concerning the relationship between venous thrombosis and VWF are contradictory [26].

The VWF is produced and secreted by endothelial cells, megakaryocytes, and platelets. The majority of VWF (80–90%) in blood plasma is produced by endothelial cells. The secretion of VWF from Weibel–Palade bodies occurs in response to endothelial-damaging factors of various etiologies. The VWF forms multimers (ultralarge von Willebrand factor, or ULvWF), the size of which is regulated by ADAMTS-13 (A disintegrin and metalloproteinase with thrombospondin-type motifs), sometimes called VWF-cleaving protease. The larger the size of the VWF multimer, the greater its functional activity. Thus, the cleavage or reduction in VWF multimers by the metalloproteinase ADAMTS-13 is essential for the maintenance of normal hemostasis. In the absence of ADAMTS-13 activity, as in patients with ADAMTS-13 mutations or acquired autoantibodies that block ADAMTS-13 activity, ULvWF “chords” with adhesive properties capture platelets and promotes thrombosis [27].

The most severe deficiency (<10% of the reference values) of ADAMTS-13 occurs in thrombocytopenic purpura, a condition characterized by large VWF multimer sizes and, consequently, an increased risk of thrombosis. Since COVID-19 is associated with a significant increase in VWF levels, there is a relative deficiency of ADAMTS-13, resulting in large VWF multimers [28]. A number of studies have reported an imbalance of VWF and ADAMTS-13 in COVID-19 patients, which is associated with a high thrombotic risk [21,29,30].

Philippe et al. showed that VWF was the best predictor of hospital mortality in COVID-19, and its serum levels correlated with the disease severity. A high level of high-molecular-weight VWF multimers in the blood of critically ill COVID-19 patients has been reported [31].

Interestingly, bacterial pneumonia and ventilator support are among factors associated with the enhanced immunostaining for VWF in the pulmonary endothelium. The increase in VWF factor in plasma, in combination with reduced level of metalloproteinase responsible for its cleavage, were found not only in COVID-19, but also in community-acquired pneumonia [32]. The ability of the most common pneumonia pathogen Streptococcus pneumoniae to induce exocytosis of Weibel–Palade bodies and VWF and interleukin 8 (IL-8) release from pulmonary endothelial cells has been reported [33].

Lung ventilation is an essential component of the comprehensive treatment of critically ill patients. At the same time, it can be associated with many complications [34]. Ventilator-induced lung injury includes barotrauma, volutrauma, and biotrauma, among others. Plasma levels of interleukin 6, interleukin-8, surfactant protein D, and soluble tumor necrosis factor receptor I/II (sTNFrI/II), as well as endothelial damage markers such as intercellular adhesion molecule-1 (ICAM-1) and VWF, have been shown to be elevated in patients with acute lung injury, and their levels change rapidly in response to different ventilation strategies [35]. Ware et al. found that VWF is an independent predictor of hospital mortality in patients with acute lung injury and ARDS, and also showed an increase in VWF in plasma, depending on the duration of lung ventilation [36]. Yiming et al. examined the interaction of platelets with endothelial cells during experimental lung ventilation with high tidal volume. The authors found that during ventilation, platelets transported the platelet-binding proteins, including VWF, to the surface of endothelial cells [37]. Hegeman et al. showed that alveolar distension developing in ventilation induced pulmonary endothelial activation in healthy mice, which was characterized by higher expression of E-selectin and VCAM-1 mRNA [38]. Pulmonary artery thrombosis was detected in a morphological study of rat lungs, 24 h after a 2 h ventilation with a flow rate of 1 L/min [34].

Thrombus formation in COVID-19 is not limited to the pulmonary vessels. This is confirmed by complications such as ischemic stroke [7] bowel ischemia [39], liver sinusoidal thrombosis [40], and disseminated intravascular coagulation [10]. Based on the results of studies confirming the increase in the VWF levels and the inhibition and/or decrease in the activity of ADAMTS-13 in COVID-19 [41], it could be that the described changes in pulmonary vascular endothelium are systemic, and the VWF plays a key role in thrombus formation in all organs. In addition, an increase in the factor in the endothelium of other organs may be due to hypoxia, which develops as a result of respiratory failure [42]. At the same time, a number of factors affect the pulmonary vascular endothelium, the influence of which is absent in other organs: baro- and volutrauma during mechanical ventilation, or inflammation associated with bacterial infection. Studies of vascular endothelium in the other organs using similar methods can be useful for providing a rationale for the role of VWF in thrombotic events in other organs.

It can be assumed that the increased intensity of IHC staining for VWF in the pulmonary vessels was the result of its enhanced multimerization and platelet adhesion to these multimers. Several studies showed the increased number of pulmonary megakaryocytes in patients with acute lung injury and ARDS of various etiologies, including COVID-19 [43,44,45]. During histological examination, we noted the presence of single megakaryocytes in the lungs, but not in all cases. Samsonova M.V. et al. showed [46] single megakaryocytes in the capillaries of the interalveolar septa in the lungs of those who died from COVID-19 in the first week of the disease. Intensity of VWF immunohistochemical staining in the endothelium according to the results of our study is noted at later stages of the disease. Nevertheless, we cannot rule out the role of megakaryocytes in the pathogenesis of pulmonary vascular thrombosis. Additional studies are required to precisely define the contribution of megakaryocytes in thrombus formation in COVID-19.

The low percentage of thrombosis early in the disease is consistent with the hypothesis of gradual depletion of the protease ADAMTS-13 in COVID-19 and its deficiency late in the disease, which leads to an increase in the size and number of VWF multimers underlying thrombus formation. Since bacterial pneumonia and lung ventilation are independent predictors of increased IHC reaction for VWF, more focus should be placed on the role of lung ventilation and superinfection in the pathogenesis of thrombus formation in COVID-19.

## 5. Conclusions

A high incidence of thrombosis, shown by increased immunostaining intensity for VWF in pulmonary vascular endothelium of patients with a disease duration of more than 10 days, confirmed the hypothesis of circulatory disorder progression during the entire period of COVID-19. The important role of VWF in thrombosis development in COVID-19 was supported by significantly more intensive VWF immunostaining in pulmonary vessel endothelium of patients with thrombotic complications than the patients without thrombotic complications. At the same time, patients with thrombosis had significantly higher VWF immunostaining intensity in the arterial, venous, and arteriolar endothelium than those with pulmonary embolism. The disease duration and thrombotic complications were found to be independent predictors of the increased intensity of immunostaining for VWF in the endothelium of pulmonary arteries, veins, and arterioles. The results of the study demonstrated an important role of endothelial VWF in the pathogenesis of pulmonary vascular thrombosis in COVID-19.

## Figures and Tables

**Figure 1:**
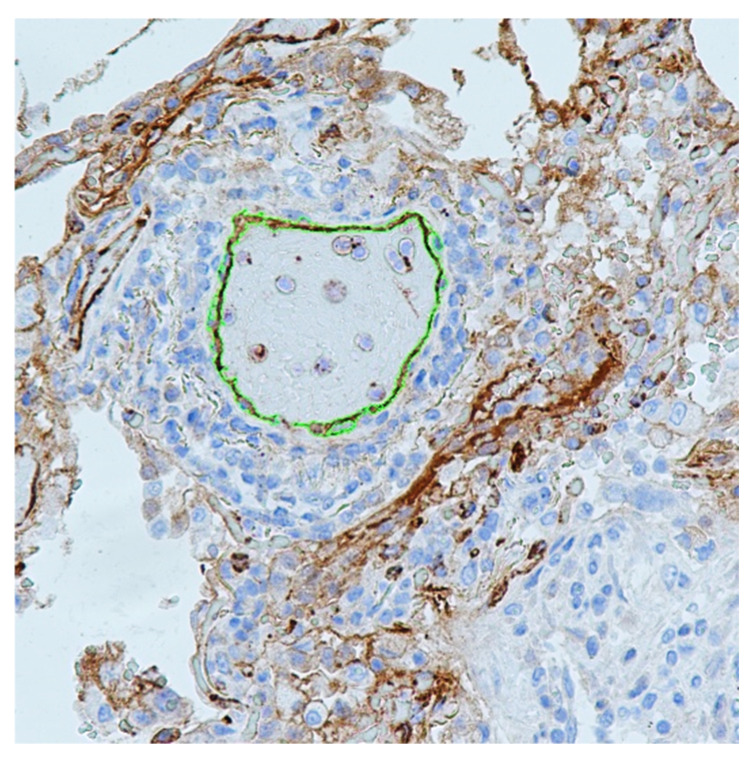
Positive immunohistochemical staining (brown) for VWF in the lungs of COVID-19 nonsurvivors. Area for assessment of staining intensity (arterial endothelium) shown in green. ×400 magnification.

**Table 1:** Frequency and structure of thrombotic complications in COVID-19 nonsurvivors in relation to disease duration.

		Disease Duration Less than 10 Days (n = 12)	Disease Duration More than 10 Days (n = 17)	*p*-Value (Fischer’s Exact Test)
**Type of thrombotic complication**	**Pulmonary** **artery embolism**	25% (3)	24% (4)	0.9
	**Pulmonary vascular thrombosis**	8% (1)	41% (7)	0.093

**Table 2:** Comparative intergroup analysis of mean optical density in pulmonary vascular endothelium in groups of patients with duration of disease more/less than 10 days.

Parameter	Disease Duration Less than 10 Days	Disease Duration 10 Days and More	*p*-Value (Mann–Whitney U-Test)
Mean optical density in arteries	0.43 (IQR, 0.38–0.50)	0.52 (IQR, 0.44–0.61)	<0.001 *
Mean optical density in veins	0.44 (IQR, 0.36–0.53)	0.52 (IQR, 0.45–0.61)	<0.001 *
Mean optical density in arterioles	0.41 (IQR, 0.36–0.47)	0.45 (IQR, 0.39–0.57)	<0.001 *
Mean optical density in venules	0.48 (IQR, 0.43–0.51)	0.51 (IQR, 0.42–0.59)	0.043 *

Note: * significant differences; IQR—interquartile range.

**Table 3:** Comparative intergroup analysis of mean optical density in pulmonary vascular endothelium in groups of patients with/without thrombotic complications.

Parameter	Patients without Thrombotic Complications	Patients with Thrombotic Complications	*p*-Value (Mann–Whitney U-Test)
Mean optical density in arteries	0.45 (IQR, 0.39–0.52)	0.48 (IQR, 0.41–0.58)	<0.001 *
Mean optical density in veins	0.46 (IQR, 0.39–0.56)	0.52 (IQR, 0.45–0.61)	<0.001 *
Mean optical density in arterioles	0.42 (IQR, 0.37–0.50)	0.43 (IQR, 0.37–0.53)	0.601
Mean optical density in venules	0.48 (IQR, 0.43–0.55)	0.51 (IQR, 0.42–0.59)	0.358

Note: * significant differences; IQR—interquartile range.

**Table 4:** Comparative intergroup analysis of mean optical density in vascular endothelium in the groups of patients with PE and thrombosis.

Parameter	Patients with PE	Patients with Thrombosis	*p*-Value (Mann–Whitney U-Test)
Mean optical density in arteries	0.46 (IQR, 0.39–0.53)	0.52 (IQR, 0.43–0.60)	<0.001 *
Mean optical density in veins	0.48 (IQR, 0.41–0.57)	0.54 (IQR, 0.47–0.62)	<0.001 *
Mean optical density in arterioles	0.40 (IQR, 0.35–0.47)	0.54 (IQR, 0.44–0.62)	<0.001 *
Mean optical density in venules	0.48 (IQR, 0.39–0.61)	0.51 (IQR, 0.46–0.59)	0.120

Note: * significant differences; IQR—interquartile range.

**Table 5:** Multivariable regression analysis: independent predictors of increased mean optical density (above 0.46) in arterial endothelium (with adjusted odds ratio calculation).

Predictor	Adjusted Odds Ratio (Multivariable Analysis)	*p*-Value
Day 10 and more	4.570 (95% CI, 3.416–6.114)	<0.001 *
Age 57 years and older	0.790 (95% CI, 0.588–1.063)	0.120
Thrombotic complications	2.519 (95% CI, 1.819–3.488)	<0.001 *
Ventilatory support	0.381 (95% CI, 0.266–0.546)	<0.001 *
Bacterial pneumonia	0.483 (95% CI, 0.361–0.647)	<0.001 *
Sex (female)	1.299 (95% CI, 0.934–1.806)	0.120

Note: * significant differences; CI—confidence interval.

**Table 6:** Results of multivariable regression analysis: independent predictors of increased mean optical density (above 0.50) in venous endothelium (with adjusted odds ratio calculation).

Predictor	Adjusted Odds Ratio (Multivariable Analysis)	*p*-Value
Day 10 and more	2.556 (95% CI, 1.781–3.669)	<0.001 *
Age 57 years and older	1.132 (95% CI, 0.824–1.555)	0.443
Thrombotic complications	1.544 (95% CI, 1.128–2.113)	0.007 *
Ventilatory support	1.062 (95% CI, 0.707–1.592)	0.771
Bacterial pneumonia	0.854 (95% CI, 0.618–1.179)	0.854
Sex (female)	3.102 (95% CI, 2.165–4.446)	<0.001 *

Note: * significant differences; CI—confidence interval.

**Table 7:** Multivariable regression analysis: independent predictors of increased mean optical density (above 0.43) in arteriolar endothelium (with adjusted odds ratio calculation).

Predictor	Adjusted Odds Ratio (Multivariable Analysis)	*p*-Value
Day 10 and more	4.188 (95% CI, 2.638–6.646)	<0.001 *
Age 57 years and older	1.074 (95% CI, 0.741–1.556)	0.706
Thrombotic complications	0.637 (95% CI, 0.423–0.958)	0.030 *
Ventilatory support	1.092 (95% CI, 0.701–1.699)	0.697
Bacterial pneumonia	0.462 (95% CI, 0.299–0.713)	<0.001 *
Sex (female)	1.433 (95% CI, 0.953–2.153)	0.084

Note: * significant differences; CI—confidence interval.

**Table 8:** Multivariable regression analysis: independent predictors of increased optical density (above 0.49) in venule endothelium (with adjusted odds ratio calculation).

Predictor	Adjusted Odds Ratio (Multivariable Analysis)	*p*-Value
Day 10 and more	1.617 (95% CI, 0.907–2.880)	0.103
Age 57 years and older	0.840 (95% CI, 0.453–1.558)	0.581
Thrombotic complications	1.196 (95% CI, 0.652–2.194)	0.563
Ventilatory support	1.153 (95% CI, 0.596–2.230)	0.672
Bacterial pneumonia	3.287 (95% CI, 1.567–6.893)	0.002 *
Sex (female)	1.843 (95% CI, 1.065–3.190)	0.029 *

Note: * significant differences; CI—confidence interval.

## Data Availability

The data that support the findings of this study are available from the corresponding author upon reasonable request. Participant data without names and identifiers will be made available after approval from the corresponding author and local Ethics Committee.

## Supplementary Materials

The following supporting information can be downloaded at: https://www.mdpi.com/article/10.3390/v14020211/s1. Table S1: Characteristics of patients included in the study.
