# Supplementary material for: The Role of Von Willebrand Factor in the Pathogenesis of Pulmonary Vascular Thrombosis in COVID-19
Source: Viruses. 2022 Jan 21;14(2):211. doi: 10.3390/v14020211 (PMC8874644; doi:10.3390/v14020211)
Supplement: Supplementary file 1 [file viruses-14-00211-s001.zip › viruses-1540563-supplementary.pdf]

Table S1. Characteristics of patients included in the study.

	Desease duration		Thrombotic complications			Age		Ventilatory support		Bacterial pneumonia		Sex	
	<10 days	>10 days	Without thrombotic complications	Pulmonary artery embolism	Pulmonary vascular thrombosis	<57	>57	Yes	No	Yes	No	Female	Male
Patient 1	×			×			×	×		×			×
Patient 2		×	×				×	×		×			×
Patient 3		×	×				×		×		×		×
Patient 4		×	×			×			×		×	×	
Patient 5		×		×			×		×		×		×
Patient 6		×	×			×			×		×		×
Patient 7	×				×		×	×			×	×	
Patient 8		×		×		×		×			×	×	
Patient 9	×		×			×			×		×		×
Patient 10	×		×			×		×			×	×	
Patient 11	×			×		×		×			×		×
Patient 12		×	×			×		×			×		×
Patient 13		×			×		×	×			×		×
Patient 14		×			×	×		×			×		×
Patient 15	×		×				×		×		×		×
Patient 16	×		×			×			×		×		×
Patient 17	×		×				×		×	×			×
Patient 18	×		×				×		×		×	×	
Patient 19		×	×			×		×		×			×
Patient 20	×			×			×		×		×	×	
Patient 21		×			×		×	×			×		×
Patient 22		×			×	×		×		×			×
Patient 23		×			×		×	×		×		×	
Patient 24		×			×		×	×			×	×	
Patient 25		×		×		×		×		×			×
Patient 26	×		×				×	×			×	×	
Patient 27	×		×			×		×			×		×
Patient 28		×			×	×		×			×		×
Patient 29		×		×			×	×		×			×
